# Giant proximity exchange and flat Chern band in 2D magnet-semiconductor heterostructures

**DOI:** 10.1126/sciadv.abn1401

**Published:** 2023-02-24

**Authors:** Nisarga Paul, Yang Zhang, Liang Fu

**Affiliations:** Department of Physics, Massachusetts Institute of Technology, Cambridge, MA, USA.

## Abstract

van der Waals (vdW) heterostructures formed by two-dimensional (2D) magnets and semiconductors have provided a fertile ground for fundamental science and spintronics. We present first-principles calculations finding a proximity exchange splitting of 14 meV (equivalent to an effective Zeeman field of 120 T) in the vdW magnet-semiconductor heterostructure MoS _2_/CrBr _3_, leading to a 2D spin-polarized half-metal with carrier densities ranging up to 10^13^ cm^−2^. We consequently explore the effect of large exchange coupling on the electronic band structure when the magnetic layer hosts chiral spin textures such as skyrmions. A flat Chern band is found at a “magic” value of magnetization $\bar{m}∼0.2$ for Schrödinger electrons, and it generally occurs for Dirac electrons. The magnetic proximity–induced anomalous Hall effect enables transport-based detection of chiral spin textures, and flat Chern bands provide an avenue for engineering various strongly correlated states.

## Teaser

Electrons coupled to spin textures or skyrmion crystals may exhibit flat Chern bands, a setting for strong correlations.​

## INTRODUCTION

Flat band materials have recently emerged as an area of intensive study in condensed matter physics (*1*–*3*). Because of the dominance of many-body effects over the quenched kinetic energy, such systems provide avenues for realizing strongly correlated electronic states such as generalized Wigner crystals, magnetic orders, and superconductivity. At partially filling of a topological flat band, interaction effects can further lead to quantum anomalous Hall states (*4*–*7*) as proposed in twisted bilayer graphene (TBG) (*8*, *9*) and transition metal dichalcogenide (TMD) moiré heterostructures (*10*–*12*). A common element of moiré materials is the presence of a spatially varying periodic modulation, e.g., of interlayer tunneling strength, which generates narrow or flat minibands.

Such a modulation is also present in magnets hosting periodic spin textures, which couple to electrons as a Zeeman splitting with spatially varying orientation. Noncoplanar or chiral spin textures, including skyrmions and canted spirals or vortices, are characterized by their nonzero spin chirality defined as ${\vec{S}}_{i}\cdot ({\vec{S}}_{j}\times {\vec{S}}_{k})$. Chiral spin textures have been the focus of recent interest due to the observation of skyrmions in a host of compounds and a large anomalous Hall effect in noncoplanar magnets (*13*–*21*). Electrons coupled to skyrmions experience an emergent magnetic field that manifests in the topological Hall effect (*22*, *23*). The presence of electron minibands due to a superlattice modulation is common to both structural moirés and periodic spin textures (*24*–*27*), but the close analogy between the two types of systems has not yet been explored.

A natural question is whether chiral spin textures can provide a route to realizing flat bands. The spatial profile of spin textures can be tuned by externally controllable parameters, such as magnetization and period, which may function similarly to twist angle or lattice mismatch in TBG or TMDs in optimizing band flatness. Moreover, the emergent magnetic field may generate minibands with Chern number (*28*), furthering the analogy with structural moirés. A possible hurdle is that the exchange coupling between localized spins and itinerant electrons must be large for emergent field to have a strong effect on the electronic band structure. In reality, however, most skyrmion materials are high density metals in which the exchange splitting is small compared to the Fermi energy.

In this work, we propose a platform for a topological flat band in two-dimensional (2D) magnet/TMD semiconductor heterostructures, in which the magnetic exchange coupling introduces an emergent Zeeman field acting on a low density of carriers in the semiconductor. Our study is motivated by the recent theoretical (*29*–*32*) and experimental works (*33*) suggesting the existence of skyrmion phases and periodic chiral spin textures arising from a spatially varying interlayer coupling in magnetic moirés such as twisted bilayers of CrI_3_ or CrBr_3_.

Our two main results are as follows. First, we predict from first-principles calculations a giant exchange splitting of 14 meV in MoS_2_/CrBr_3_, which far exceeds the values found so far in 2D TMD/magnet heterostructures. As a result of this large exchange splitting, for a wide range of hole densities up to 0.83 × 10^13^cm^−2^, only one spin-polarized band is occupied, leading to a 2D half-metal. Second, we use a continuum model approach to study the effect of the emergent field from magnetic skyrmion crystals on the low-energy electronic structure of proximitized 2D semiconductors. We find an almost completely flat Chern band for Schrödinger electrons (as in MoS_2_) at a “magic” value of magnetization $\bar{m}=0.2$, while for Dirac electrons (as in graphene), a flat Chern band always occurs.

## RESULTS

### Giant exchange splitting in MoS_2_/CrBr_3_

Our proposal to realize flat Chern bands requires a strong interfacial exchange coupling in 2D magnet-TMD semiconductor heterostructures. In monolayer TMDs, strong Ising spin-orbit coupling and inversion symmetry breaking gives rise to opposite spin states in the *K*^′^ and *K* valleys. Previous studies have demonstrated *K*^′^/*K* spin-valley splitting in TMDs proximity-coupled to ferromagnetic semiconductors. However, such splitting is generally small, e.g., 3.5 meV in WSe_2_/CrI_3_ (*34*, *35*).

We performed density functional calculations using the generalized gradient approximation (*36*) with SCAN+rVV10 van der Waals (vdW) density functional (*37*), as implemented in the Vienna Ab initio Simulation Package (*38*). By scanning through various 2D TMD-magnet heterostructures, we find a giant exchange splitting in MoS_2_/CrBr_3_, where monolayer MoS_2_ is a TMD semiconductor and CrBr_3_ is an insulating vdW magnet. Magnetic orders and lattice constants are taken from the Computational 2D Materials Database (*39*). The 2 × 2 supercell of MoS_2_ with lattice constant of 6.38 Å is chosen to match the lattice constant of CrBr_3_ (6.44 Å) with less than 1% strain. Similar to previous works, we find a small *K*^′^/*K* valley splitting on the valence band side of 0.9 meV for MoS_2_/CrBr_3_.

**Fig. 1.**
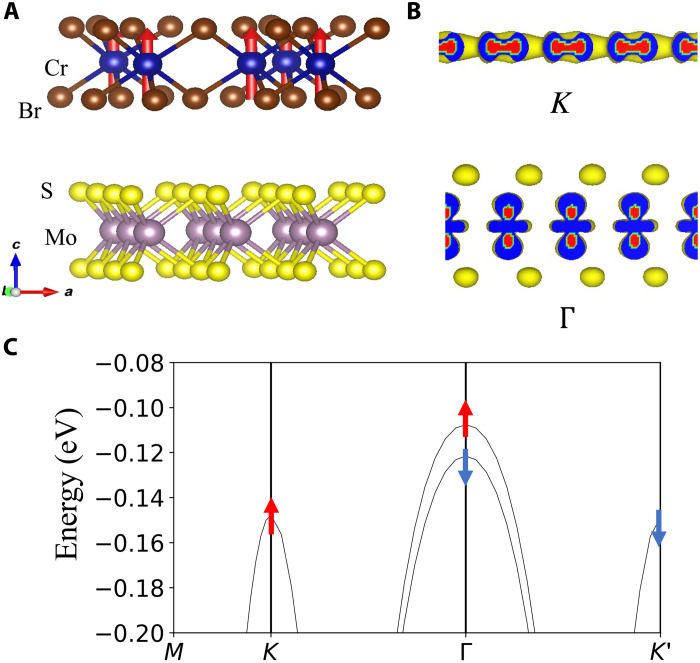
The large exchange splitting in MoS_2_/CrBr_3_as a route to flat Chern bands. (**A**) Lattice structure and magnetic order of MoS_2_/CrBr_3_ heterostructures. (**B**) Kohn-Sham wave function for *K* valley and Γ valley maximum. The *K* valley wave function is localized in the Mo layer, while the Γ valley wave function is spread out in the *z* direction. (**C**) Band structure of the fully relaxed MoS_2_/CrBr_3_ heterostructure with a 0.9-meV valley splitting at *K*^′^/*K* and 14-meV spin splitting at Γ.

In monolayer MoS_2_, the Γ valley is very close in energy to the *K* valley. In MoS_2_/CrBr_3_, we find that the Γ valley of MoS_2_ becomes the valence band maximum after taking into account the relaxed heterostructure with a layer spacing of 6.76 Å (defined as the distance between the center of MoS_2_ and CrBr_3_). We observe a giant spin splitting of up to 14 meV in the Γ valley as shown in Fig. 1. Assuming a Landé *g*-factor equal to 2, this is an effective Zeeman field of 120 T. (For MoS_2_ on twisted bilayer CrBr_3_, which is relevant for skyrmion physics, we find a similarly giant spin splitting of 17 meV; see the Supplementary Materials.) The large difference in the exchange splitting in the *K* and Γ valleys can be understood from the spatial distribution of wave functions. At the *K* valley, because of the large in-plane momentum, electron wave functions are strongly localized within the vdW layer. However, at the Γ valley, because of the zero in-plane momentum, the wave functions are more spread out in out-of-plane direction, which enhances coupling to the magnetic layer. The large exchange splitting in this specific material, which is the first main result of this work, provides efficient spin control of the Γ valley bands.

### Emergent gauge field at large exchange coupling

In the following, we explore the consequences of a large exchange coupling on the electronic band structure. This is motivated by both the giant exchange splitting found in the previous section and theoretical and experimental results suggesting that the twisted bilayers of vdW magnets may host noncoplanar or skyrmionic phases (*29*–*33*). To this end, we turn to a completely general Hamiltonian describing electrons coupled to a spin texture via a large exchange coupling. Note that, in low density systems such as TMD semiconductors or graphene, the physics of itinerant electrons coupled to periodic spin textures is universally described by a continuum theory, taking the form

$$H_{S}=\frac{p^{2}}{2m}+J\vec{\sigma }\;\cdot \vec{S}(r)$$ (1A)

$$H_{D}=v\vec{p}\;\cdot \vec{\tau }+J\vec{\sigma }\;\cdot \vec{S}(r)$$ (1B)

for the case of Schrödinger and Dirac electrons with mass *m* and velocity *v*, respectively. The Pauli $\vec{\sigma }$ matrices act on electron spin, while the Pauli $\vec{\tau }$ matrices act on a psuedospin degree of freedom, e.g., sublattice in the case of graphene. Equation 1A describes TMD semiconductors proximity-coupled to a magnetic layer, while Eq. 1B describes Dirac materials such as graphene and TBG.

In the following, we will consider smoothly varying periodic spin textures $\vec{S}(r)$ with magnetic wavelength ξ and unit norm $|\vec{S}|=1$. An SU(2) gauge transformation **U**(*r*) allows us to instead consider a uniformly polarized spin texture provided that we use the gauge-transformed momentum $\vec{p}1-e\vec{A}$, where

$$\vec{A}=\frac{i\hbar }{e}U^{†}\vec{\nabla }U$$ (2)

The large exchange coupling limits are, respectively

$$Jm\xi^{2}\gg 1\;\mathrm{and}\;J\xi /v\gg 1$$ (3)

In this limit, the ground state manifold consists of electrons with spins locally anti-aligned with the spin texture. The effective Hamiltonian describing the locally spin-polarized Schrödinger electrons has been derived before (*22*)

$${\tilde{H}}_{S}=\frac{1}{2m}{\left(\vec{p}-e\vec{A}\right)}^{2}+\frac{e^{2}}{8m}{\left(\partial_{i}\vec{S}\right)}^{2}$$ (4)

(summing *i* = *x*, *y*). The second term, originating from off-diagonal terms of $\vec{A}$, is a scalar potential that accounts for the reduction of electron hopping by the spin gradient and $\vec{A}={\left(\vec{A}\right)}_{↓↓}$ is an emergent *U*(1) gauge field. The corresponding emergent field strength

$$B^{e}=\frac{\hbar }{2e}\vec{S}\;\cdot \;(\partial_{x}\vec{S}\times \partial_{y}\vec{S})$$ (5)

is proportional to the spin chirality. A skyrmion can be defined as a texture with flux *h*/*e*. It follows from this equation that purely in-plane spin textures have vanishing emergent field. Moreover, purely in-plane textures are symmetric under the combination of time reversal and a spin-space *C*_2_ rotation, while the Chern number is odd under this combination, implying that such textures do not yield topological bands. Accordingly, we will consider chiral magnets with out-of-plane spin textures.

We will also be interested in Dirac electrons at large exchange coupling, for which the effective Hamiltonian takes the form

$${\tilde{H}}_{D}=v(\vec{p}-e\vec{A})\;\cdot \vec{\tau }$$ (6)

It is notable that Dirac electrons only feel a periodic gauge field in contrast with the previously studied Schrödinger case involving both the gauge field and a scalar potential.

The origin of the emergent gauge field is the local U(1) rotational freedom of the electron spin about the axis of the local spin. In analogy with conventional Landau levels, the emergent field *B^e^* generally endows the minibands with Chern number when the average is nonzero.

In the following, we investigate the flatness of the induced Chern bands in the large exchange coupling limit. How realistic is the large exchange coupling limit for the systems that we have in mind? Our primary motivation is MoS_2_/CrBr_3_, for which *J* ≈ 7 meV and m^*^ ≈ 1.1*m_e_*. Moiré magnets can exhibit periods ξ of 10 to 100 nm (*29*, *40*, *41*). Together (restoring ℏ), we have $\frac{Jm\xi^{2}}{\hbar^{2}}∼10\;\mathrm{to}\;10^{3}$ for this system, i.e., we are deep into the large exchange limit for this range of ξ, although theoretical estimates for skyrmion sizes in CrBr_3_ are only approximate.

When ξ is large, the kinetic energy scale ℏ^2^/*m*ξ^2^ is small, and disorder may have a pronounced effect. However, all the low-lying bands are topological (*C* = 1) bands, so a substantial anomalous Hall effect is still expected (*28*).

For monolayer graphene, assuming *J* ∼ 1 meV and *v* = 2.5 · 10^6^ m/s, *J*ξ/ℏ*v* ∼ 0.4. However, the velocity can be sharply decreased in TBG, and ξ increased in a moiré magnet, so the large exchange limit can be achieved for Dirac materials as well. The large exchange limit is closely related to the adiabatic limit that has been frequently studied in the literature (*42*–*44*). A key difference is that our criterion makes no reference to electron density or scattering time, because we are working in the limit of pristine samples and low densities on the order of one charge per SkX unit cell.

### Flat bands in skyrmion crystals

We present numerical results on the band structure of Schrödinger and Dirac electrons in the presence of generic skyrmion crystals using the full Hamiltonians in Eqs. 1A and 1B, i.e., without any approximations. *H*_S_ and *H*_D_ are explicitly diagonalized in a plane wave basis with a large wave number cutoff *N* (see the Supplementary Materials).

**Fig. 2.**
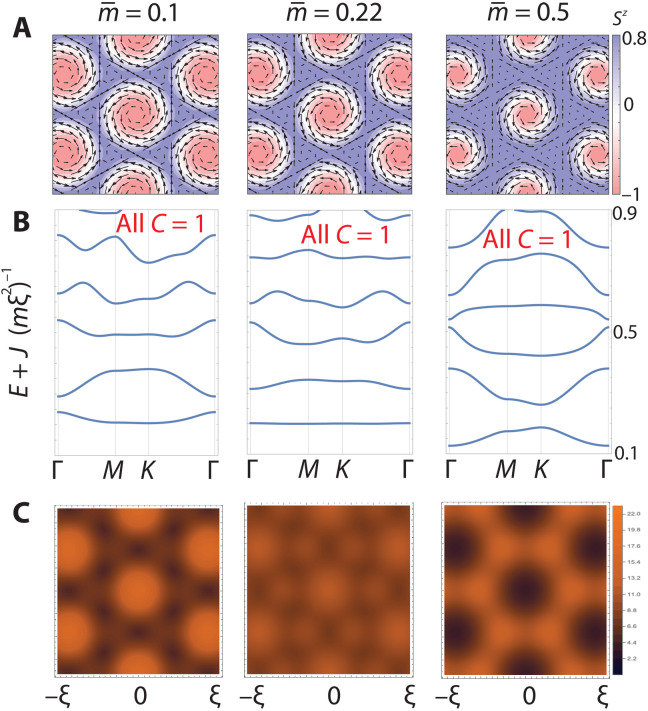
Flat Chern band from a skyrmion crystal: Schrödinger electrons. (**A**) Skyrmion texture based on generic ansatz (Eq. 7) with different magnetizations and α = 1. (*S^x^*, *S^y^*) is plotted as a vector field with *S^z^* in color, and $\bar{m}$ is the average of *S^z^*. (**B**) Band structures in the presence of the textures in (A), following the Γ*MK*Γ path in the hexagonal mini-Brillouin zone. *H*_S_ was diagonalized using a plane wave cutoff *N* = 10, taking *J* = 100, *m* = 1. A flat first band is observed when $\bar{m}=0.22$. All bands carry Chern number *C* = 1. (**C**) Local density of states (LDOS) summed over the first band (sampling 4^2^ states), spatially aligned with (A).

As a model for a generic SkX, we introduce the simple three-parameter ansatz $\vec{S}={\vec{N}}_{r}/|{\vec{N}}_{r}|$ with

$${\vec{N}}_{r}=\frac{1}{\sqrt{2}}\sum_{j=1}^{6}\;e^{iq_{j}\cdot r}{\hat{e}}_{j}+\mu \hat{z}$$ (7)

where ${\vec{q}}_{j}=\xi (\cos\theta_{j},\sin\theta_{j})$ and ${\hat{e}}_{j}=(i\alpha \sin\theta_{j},-i\alpha \cos\theta_{j},-1)/\sqrt{2}$ and the angles satisfy θ_2_ = θ_1_ + 2π/3, θ_3_ = θ_1_ + 4π/3, and θ_*j*+3_ = θ*_j_* + π. Equation 7 represents a normalized sum of three helical spirals forming a triangular SkX, as plotted in Fig. 2A. This ansatz is widely adopted in studies of chiral magnets and magnetic skyrmion crystals. It qualitatively reproduces the observed real-space images of skyrmion crystals (*45*–*51*). A different choice of relative phases results in Néel-like skyrmions but does not alter band structures. The parameters α, ξ, and μ control coplanarity, wavelength, and out-of-plane bias, respectively.

In Fig. 2, we plot band structures and local density of states (LDOS) for Schrödinger electrons in the presence of our chosen SkX. We set α = 1 and vary the magnetization $\bar{m}$ (i.e., the unit cell average of *S^z^*), which is monotonic with μ (ξ can be scaled out of the problem). We can observe the flattening of lower bands and a near perfectly flat, well-separated first band as we tune the magnetization past $\bar{m}=0.2$, the second main result of this work. All bands shown carry Chern number *C* = 1, and spins are anti-aligned with the skyrmion texture.

**Fig. 3.**
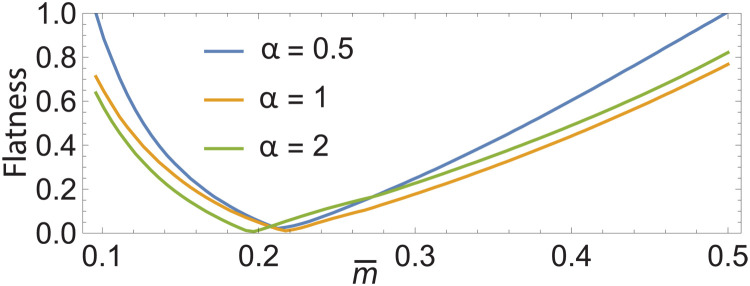
Flatness at a special magnetization. First band flatness, defined as the ratio of bandwidth to bandgap, of *H*_S_ in the presence of a generic skyrmion crystal (defined in Eq. 7) with varying magnetization $\bar{m}$ and coplanarity α. Near-perfect flatness occurs around $\bar{m}=0.2$. Flatness was calculated by solving *H*_S_ with plane wave cutoff *N* = 10 and *J* = 100 over 10^2^ points in the mini-BZ.

In Fig. 3, we systematically plot band flatness, defined as the ratio of bandwidth to bandgap for the first miniband, with varying α and $\bar{m}$. Complete flatness is an ideal pathway for strongly correlated matter because the kinetic energy is strongly quenched and band separation remains large. We plot the branch $\bar{m}>0$ that corresponds to the typical case of separated skyrmion bubbles. The plot indicates a quite robust magic value of magnetization near $\bar{m}=0.2$ where flatness goes to zero.

**Fig. 4.**
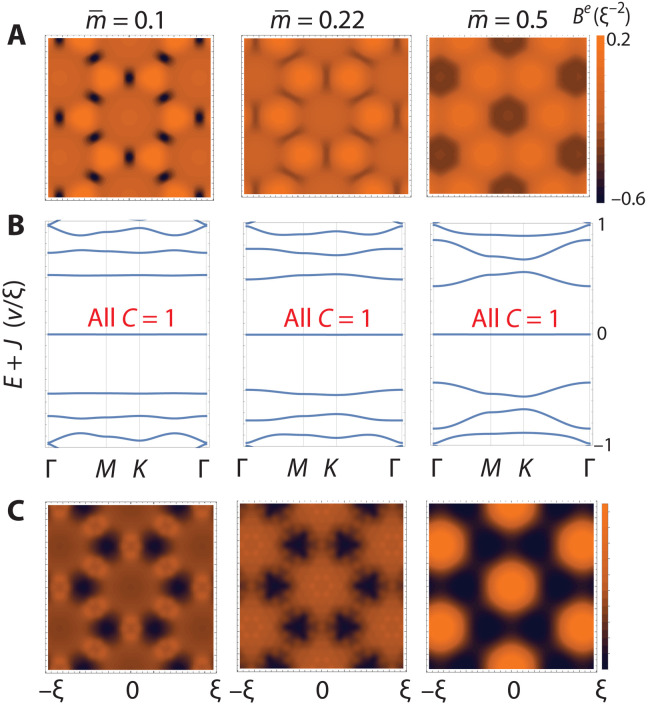
Flat Chern band from a skyrmion crystal: Dirac electrons. (**A**) Emergent magnetic fields corresponding to (and spatially aligned with) the SkX shown in Fig. 2A. Dirac electrons in the large exchange coupling limit move in this emergent field. (**B**) Band structures analogous to Fig. 2B for Dirac electrons. A flat band is pinned at *E* = − *J* whenever there is net emergent flux. All bands carry Chern number *C* = 1. (**C**) LDOS summed over the *E* = − *J* band (sampling 150 states), spatially aligned with (A).

Next, we turn to the case of Dirac electrons under periodic Zeeman field, which may be realized in graphene or TBG in proximity with a 2D magnet. We observe a flat band for arbitrary skyrmion textures in Fig. 4. This can be understood from the effective Hamiltonian in the large *J* limit

$${\tilde{H}}_{D}=v\left(\begin{array}{cc} 0 & \text{Π}\\ \bar{\text{Π}} & 0 \end{array}\right)$$ (8)

where $\bar{\text{Π}}=({\text{Π}}_{x}+i{\text{Π}}_{y})/2$ and $\vec{\text{Π}}=\vec{p}-e\vec{A}$. It is known that the Dirac equation in the presence of a spatially varying magnetic field admits an extensive set of zero-mode solutions (*52*). The number of zero modes is equal to the total number of flux quanta through the system (which, in our case, is given by the total topological charge of the skyrmion crystal), regardless of the spatial profile of the magnetic field. This remarkable property follows from the Atiyah-Singer index theorem. In the limit of a uniform field, these zero modes reduce to the well-known *n* = 0 Landau level.

In Fig. 4A, we plot the periodic emergent magnetic fields arising from the skyrmion crystal, and, in Fig. 4C, we plot the LDOS of the flat band of Dirac electrons under magnetic proximity effect. The LDOS shows significant spatial variation, which can be detected by scanning tunneling microscopy.

In real magnetic moiré systems, there may be magnetic disorder or a lack of strict periodicity in the magnetic domains (*41*). A general expectation is that the lack of a strict periodicity would broaden the band structure, which were all calculated for pristine samples. Still, we may expect a DOS enhancement even with disorder. Moreover, Dirac flat band is actually immune to magnetic disorder, and an extensive set of zero modes would exist even in the absence of periodicity. For the flat band, the only requirement is a net skyrmion density; one Dirac zero modes will bind to each skyrmion, and we expect the strongly correlated physics associated with a DOS enhancement to survive disorder in this case.

### Small exchange coupling and anomalous Hall effect

While the large exchange coupling regime is realistic for MoS_2_/CrBr_3_, other systems may have constraints on magnetic wavelength or intrinsic exchange coupling not conducive to this limit. In addition, many chiral magnets host noncoplanar states that do not enclose a net spin chirality. Examples of such states include the canted vortex lattice, multiple-Q conical spiral, multilayers of single-Q helices, and meron-antimeron lattice (*31*, *32*).

**Fig. 5.**
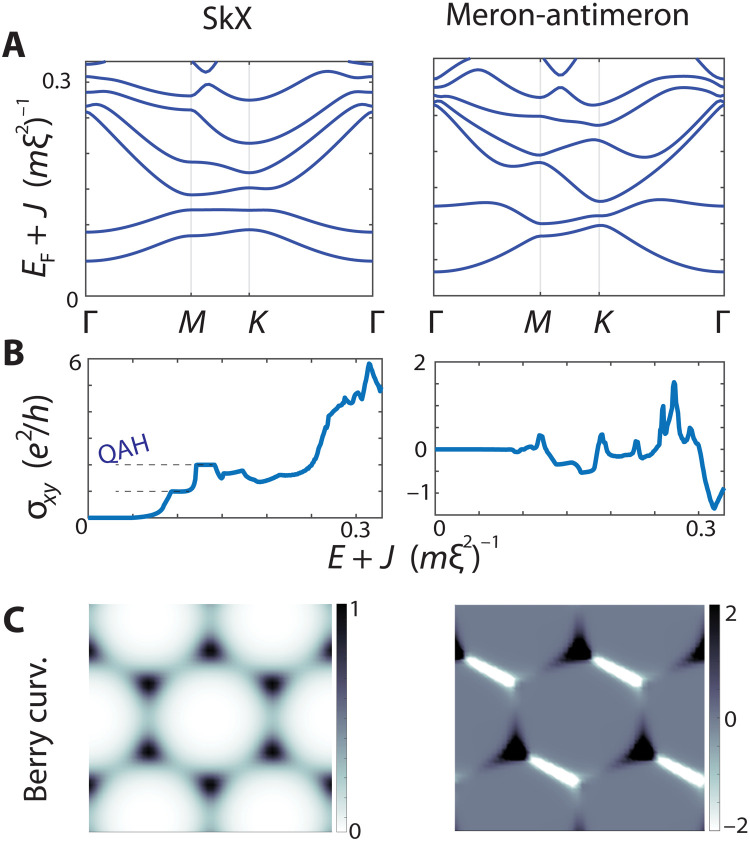
Band topology at weak coupling. (**A**) Weak-coupling band structures for the SkX ($\bar{m}=0.1,\alpha =1$) and the meron-antimeron lattice. Each band in the former has Chern number 1, and the lowest bands in the latter have Chern numbers [0, 0, 1, −1, 1]. (**B**) Hall conductances. The SkX exhibits a quantized anomalous Hall effect from the lowest bands, and the meron-antimeron lattice has a nonzero anomalous Hall response despite its vanishing net spin chirality. (**C**) Similarly for an SkX (left) and meron-antimeron lattice (right) at weak exchange coupling, corresponding to the lowest bands of (A).

In the large exchange coupling regime, we found well-separated flat Chern bands (which imply a quantized anomalous Hall effect) in the presence of skyrmion-like textures. If we drop these two prerequisites and instead consider small exchange coupling and general noncoplanar textures (i.e., not necessarily enclosing a net spin chirality), then we find numerically that an anomalous Hall conductance is still generically present. We illustrate this point with an SkX and a meron-antimeron lattice at small exchange coupling (Fig. 5A), which takes the form

$${\vec{S}}_{r}=(\sqrt{1-{\left(S^{z}\right)}^{2}}\sin\theta_{r},\sqrt{1-{\left(S^{z}\right)}^{2}}\cos\theta_{r},S^{z})$$ (9)

where θ*_r_* is the angle relative to the unit cell center and *S^z^* is a smooth periodic function that equals unity at the unit cell centers. We chose $S^{z}(r)=\frac{1}{3}\sum_{i}\cos^{2}(q_{i}\;\cdot \;r/2)$, where *q*_1_ = *b*_1_, *q*_2_ = *b*_2_, *q*_3_ = − *b*_1_ − *b*_2_, and *b*_1,2_ are reciprocal lattice vectors. This texture has alternating regions of ±1/2 emergent flux, justifying the name meron-antimeron lattice (and has only threefold symmetry). Because the net spin chirality vanishes, naïvely, we may expect no momentum-space topology. However, we observe miniband Chern numbers and a corresponding anomalous Hall effect (Fig. 5B). Moreover, we have produced plots of the Berry curvatures of the SkX and meron-antimeron lattice at weak exchange couplings (Fig. 5C).

**Fig. 6.**
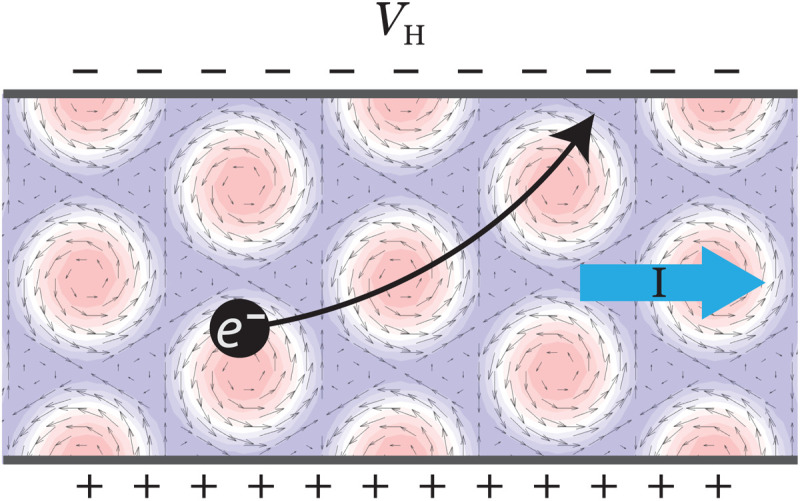
Anomalous Hall effect from real-space topology. An anomalous Hall effect is generically expected in semiconductor/chiral magnet heterostructures with noncoplanar textures even at small coupling and without net spin chirality. *V*_H_ is Hall potential and I is current.

The observation of marked Hall transport features suggests that proximity-coupled electrons can serve as a reliable sensor for noncoplanar textures in magnetic materials (Fig. 6). A transport-based sensor would be advantageous because it can be done on tabletop while bulk probes such as Lorentz transmission electron microscopy and neutron diffraction have a low sensitivity for 2D devices. We also note that, in the presence of a point-group symmetry, the leading Fourier modes of the spin texture may be inferred from the miniband Chern numbers. This provides a useful direct mapping between real-space textures and transport properties. For instance, in the presence of sixfold symmetry, the lowest band’s Chern number satisfies

$$C\;\mathrm{mod}\;6=\{\begin{array}{cc} 1 & S_{0}^{z}>0,S_{G}^{z}>0\\ 2\;\mathrm{or}\;0 & S_{0}^{z}>0,S_{G}^{z}<0\\ -2\;\mathrm{or}\;0 & S_{0}^{z}<0,S_{G}^{z}>0\\ -1 & S_{0}^{z}<0,S_{G}^{z}<0 \end{array}$$ (10)

assuming *J* > 0. For *J* < 0, the Chern number changes sign mod 6. Here, ${\vec{S}}_{0}\;\mathrm{and}\;{\vec{\;S}}_{G}$ are the zeroth and first Fourier components of $\vec{S}(r)$, so, in particular, $S_{0}^{z}=\bar{m}$. Here, **G** is one of the primitive reciprocal lattice vectors (${\vec{S}}_{G}$ does not depend on which one, by sixfold symmetry). For fixed $S_{G}^{z}$, it follows from the above that the first band undergoes a topological transition as the magnetization changes sign, which can be traced to the gap closing at Γ. Likewise, for fixed magnetization, there is a topological transition as $S_{G}^{z}$ changes sign. We leave the derivation to the Supplementary Materials.

## DISCUSSION

We investigated the emergence of flat bands in semiconductors proximity-coupled to spin textures in the limit of large exchange coupling and emerged with a concrete prediction that the TMD/vdW magnet heterostructure MoS_2_ proximity-coupled to twisted bilayer CrBr_3_ should exhibit a flat Chern band at magnetization $\bar{m}∼0.2$. Our study was motivated by the giant exchange of 14 meV (corresponding to a large exchange parameter *Jm*ξ^2^/ℏ^2^ ∼ 10^3^) that we found using first-principles calculations in MoS_2_/CrBr_3_, the latter of which is proposed to host skyrmions in a moiré (*29*, *30*). For Schrödinger electrons coupled to a generic SkX, we find a flat Chern band emerges at out-of-plane magnetization $\bar{m}=0.2$, while a flat Chern band is generically present for Dirac electrons. A large exchange coupling and long electron mean free path (for sharply defined minibands) are prerequisites for this route to flat bands. These continuum results are universal at low densities, only making use of effective mass or Dirac velocity in the band structure. We show explicitly that anomalous Hall effect is generically present in TMD semiconductors or graphene proximitized by noncoplanar magnets. Our results also suggest the possibility of fractional Chern insulators in 2D magnet-semiconductor or graphene heterostructures.

## MATERIALS AND METHODS

### Density functional theory

We performed the density functional calculations using generalized gradient approximation (*36*) with SCAN+rVV10 vdW density functional (*37*), as implemented in the Vienna Ab initio Simulation Package (*38*). Pseudopotentials are used to describe the electron-ion interactions. We first constructed 0°-aligned 2 × 2 MoS_2_/CrBr_3_ heterobilayer with vacuum spacing larger than 20 Å to avoid artificial interaction between the periodic images along the *z* direction. Dipole corrections were added to the local potential to correct the errors introduced by the periodic boundary conditions in out-of-plane direction. The structure relaxation was performed with force on each atom less than 0.001 eV/A. We used a dense *k*-mesh sampling up to 12 × 12 for structure relaxation and self-consistent calculation. In the Supplementary Materials, we detail density functional theory (DFT) calculations with the addition of a Hubbard *U* and for MoS_2_ on bilayer CrBr_3_ and find that our main conclusions are unchanged.

### Band structure

The plane wave method was used for Figs. 2 to 4, while, for Fig. 5, a square lattice nearest neighbor tight-binding Hamiltonian with parabolic low-energy dispersion was used. In the plane wave approximation (which we detail in the Supplementary Materials), we kept the 21^2^ lowest-lying Fourier modes of the skyrmion texture and electronic wave functions.

## Supplementary Materials

### This PDF file includes:

Band structure Small exchange coupling and anomalous Hall effect Berry curvature Figs. S1 to S4 References
