# Supplementary material for: Giant proximity exchange and flat Chern band in 2D magnet-semiconductor heterostructures
Source: Sci Adv. 2023 Feb 24;9(8):eabn1401. doi: 10.1126/sciadv.abn1401 (PMC12488001; doi:10.1126/sciadv.abn1401)
Supplement: Supplementary file 1 — Band structure Small exchange coupling and anomalous Hall effect Berry curvature Figs. S1 to S4 References [file sciadv.abn1401_sm.pdf]

Science Advances
AAAS

Supplementary Materials for

Giant proximity exchange and flat Chern band in 2D magnet-semiconductor heterostructures

Nisarga Paul *et al.*

Corresponding author: Liang Fu, liangfu@mit.edu; Nisarga Paul, npaul@mit.edu

Sci. Adv. **9**, eabn1401 (2023)
DOI: 10.1126/sciadv.abn1401

This PDF file includes:

Band structure
Small exchange coupling and anomalous Hall effect
Berry curvature
Figs. S1 to S4
References

Supplemental Material

Bandstructure

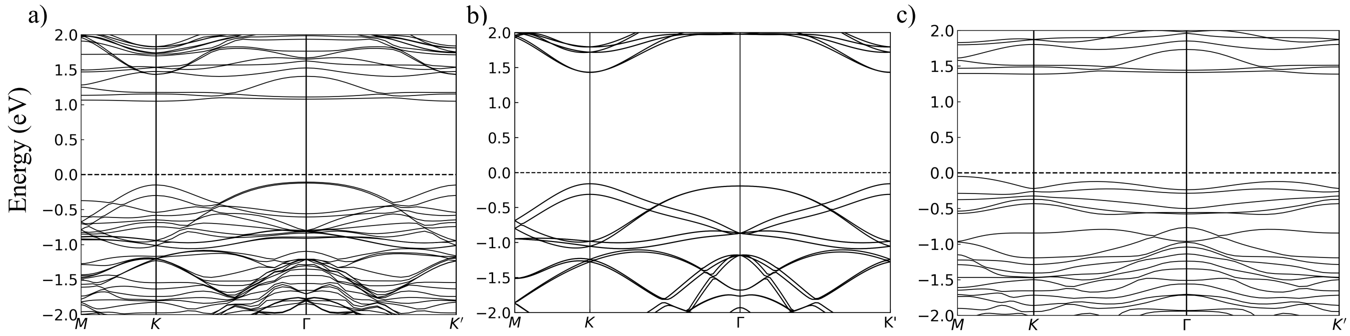

FIG. S1. **DFT bandstructures.** DFT band structure of (a) $MoS_2/CrBr_3$, (b) 2×2 MoS_2, (c) monolayer $CrBr_3$ from $E_f - 2$eV to $E_f + 2$eV. The valence band of $CrBr_3$ is only 0.4 eV lower than the valence band of $CrBr_2$.

DFT calculations. We present the separate band structures of 2×2 MoS_2 and $CrBr_3$ in Fig. S1. The K valley is 30 meV higher than the Γ valley in monolayer MoS_2. Due to the type II band alignment, the band gap of the heterobilayer is reduced to 1.2 eV. To consider the effect of Hubbard corrections, we add various values of onsite U to the present calculations with simplified (rotationally invariant) GGA + U approach [53], as implemented in the Vienna Ab initio Simulation Package [38]. We find the valence bands of $CrBr_3$ move slightly under Hubbard corrections, and the proximitized spin splitting is mainly determined by the valence band energy offset between $CrBr_3$ and MoS_2. In practice, U=1.5 eV is used in most of the recent works on CrX_3 (X=Cl, Br, I) family of materials [54], and the spin splitting at Γ valley is slightly reduced to 11 meV as shown in Fig. S3, within the strong coupling regime.

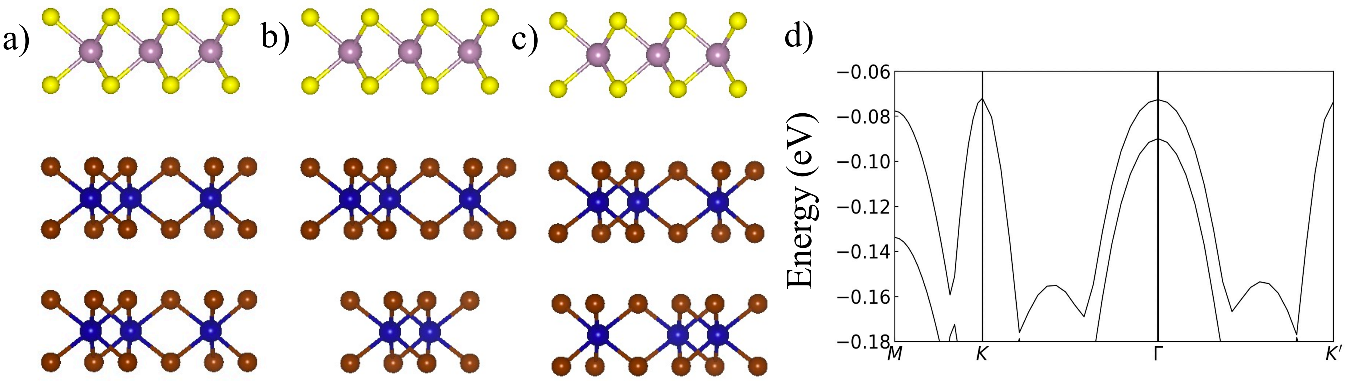

FIG. S2. **MoS_2 on bilayer $CrBr_3$.** (a,b,c) Lattice structures of of $MoS_2/CrBr_3/CrBr_3$ at high symmetry stacking regions, (d) Band structure of $MoS_2/CrBr_3/CrBr_3$ for stacking configuration (b).

Unlike the K valley wavefunction, which only couples to the out-of-plane magnetization, here the Γ valley wavefunction at zero momentum is isotropic as shown in Fig. S3 and couples to magnetization at all directions. In DFT calculations, we check the value of proximity-induced spin splitting gap both at out-of-plane and in-plane directions and find the same value as 14 meV. We further note the small directional variations in the exchange coupling will not affect our main results.

As shown in previous work [29], Skyrmion spin textures can be realized in twisted bilayer $CrBr_3$. Therefore we further calculate the exchange splitting of MoS_2 on twisted bilayers $CrBr_3$. In bilayer $CrBr_3$, the bonding-antibonding splitting within valence bands pushes valence band maximum higher than monolayer $CrBr_3$, and the valence band offset between Γ valley of MoS_2 and magnetic layers is threfore reduced. With setup $MoS_2/CrBr_3/CrBr_3$, the exchange splittings are found to be around 17 meV for three high symmetry stacking configurations in Fig. S2(a,b,c), slightly enhanced compared to the 14 meV splitting identified in $MoS_2/CrBr_3$.

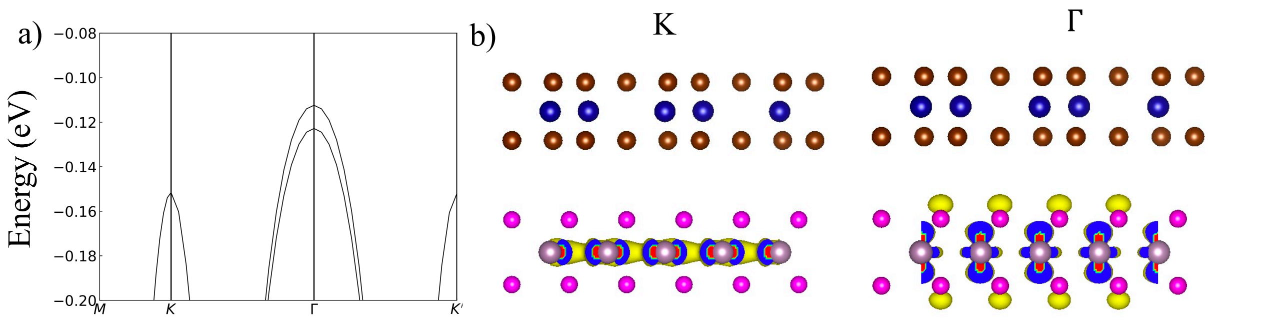

FIG. S3. **Adding a Hubbard** U**.** (a) GGA + U band structures of ferromagnetic heterobilayer $CrBr_3$ and MoS_2. A rotationally invariant Hubbard potential is employed with U = 1.5 eV in the GGA + U calculation. (b)Lattice structure of $MoS_2/CrBr_3$ with wavefunction of MoS_2 valence band at Γ and K momentum.

Continuum model. The plane wave approximation for the continuum model is as follows. Let $\vec{S}(r) = \vec{S}(r+a_1) = \vec{S}(r+a_2)$ be a doubly periodic spin texture and let b_1, b_2 be the reciprocal vectors, $b_i \cdot a_j = 2\pi\delta_{ij}$. We can write any wavefunction with momentum $k = (k_x, k_y)$ as

$$\psi(r) = e^{ikr} \sum_{m_1,m_2} e^{i(m_1 b_1 + m_2 b_2)r} \chi_{m_1 m_2} \tag{11}$$

where m_1, m_2 are integers and $\chi_{m_1 m_2} = \begin{pmatrix} \chi^{\uparrow}_{m_1 m_2} \\ \chi^{\downarrow}_{m_1 m_2} \end{pmatrix}$. The Hamiltonian can be written as

$$H_S = \frac{p^2}{2m}\mathbf{1} + J \sum_{m_1,m_2} e^{i(m_1 b_1 + m_2 b_2)r} \vec{S}_{m_1 m_2} \cdot \vec{\sigma} \tag{12}$$

where $\vec{S}_{m_1 m_2} = \vec{S}^*_{-m_1,-m_2}$ is a Fourier component of $\vec{S}(r)$. We then obtain the matrix equation

$$\frac{(k + n_1 b_1 + n_2 b_2)^2}{2m} \chi_{n_1 n_2} + J \sum_{m_1 m_2} \vec{S}_{m_1 m_2} \cdot \vec{\sigma} \chi_{n_1 - m_1, n_2 - m_2} = E \chi_{n_1 n_2} \tag{13}$$

We apply a large momentum cutoff $|n_1|, |n_2| \leq N$ to approximate the spectrum and eigenfunctions, with an analogous procedure for the Dirac case. In the Dirac case we omit spurious modes which appear on the lattice edges and whose support is measure zero as $N \to \infty$, which can be easily identified as they break the C_6 symmetry. The plane wave method was used for Fig.s 1, 3, and 4 while for Fig. 5 a square lattice nearest neighbor tight-binding Hamiltonian was used.

Small exchange coupling and anomalous Hall effect

In the following, we connect the leading Fourier modes of point-group symmetric spin textures to the Chern number of associated electron bands. We approach this by using perturbation theory in a small exchange coupling J, in which case Chern numbers can be computed from the point-group symmetry eigenvalues of the states at the high-symmetry points in the Brillouin zone [55]. These eigenvalues in turn depend only on the leading Fourier modes of the spin texture. We assume a six-fold rotational symmetry of the Hamiltonian, i.e. $[\hat{C}_6, H] = 0$ where $\hat{C}_6 = e^{\frac{-2\pi i}{6} L^z} e^{\frac{-2\pi i}{6} \frac{\sigma^z}{2}}$ (other point-groups can be analyzed similarly). The Chern number C is given by

$$e^{2\pi i C/6} = \prod_{i \in occ.} (-1)^F \eta_i(\Gamma) \theta_i(K) \zeta_i(M) \tag{14}$$

where $\eta(\Gamma)$ is the C_6 eigenvalue at the Γ point, $\theta(K)$ is the C_3 eigenvalue at the K point, and $\zeta(M)$ is the C_2 eigenvalue at the M point. The Chern number for the first band follows from degenerate perturbation theory, and we obtain

$$C \bmod 6 = \begin{cases} 1 & S_0^z > 0, S_{\mathbf{G}}^z > 0 \\ 2 \text{ or } 0 & S_0^z > 0, S_{\mathbf{G}}^z < 0 \\ -2 \text{ or } 0 & S_0^z < 0, S_{\mathbf{G}}^z > 0 \\ -1 & S_0^z < 0, S_{\mathbf{G}}^z < 0 \end{cases} \tag{15}$$

assuming $J > 0$. For $J < 0$ the Chern number changes sign mod 6. Here $\vec{S}_0, \vec{S}_{\mathbf{G}}$ are the zeroth and first Fourier components of $\vec{S}(r)$, so in particular $S_0^z = \overline{m}$. Here $\mathbf{G}$ is one of the primitive reciprocal lattice vectors ($\vec{S}_{\mathbf{G}}$ doesn't depend on which one, by six-fold symmetry). For fixed $S_{\mathbf{G}}^z$, it follows from the above that the first band undergoes a topological transition as the magnetization changes sign, which can be traced to the gap closing at Γ. Likewise, for fixed magnetization there is a topological transition as $S_{\mathbf{G}}^z$ changes sign.

Γ point.—The two states $|\Gamma, +\frac{1}{2}\rangle$ and $|\Gamma, -\frac{1}{2}\rangle$ have $\hat{C}_6$ eigenvalues $e^{-i\pi/6}$ and $e^{i\pi/6}$ respectively. First order perturbation theory gives an energy correction

$$\langle \Gamma, s | J\vec{\sigma} \cdot \vec{S} | \Gamma, s \rangle = 2sJS_0^z \tag{16}$$

where $s = \pm\frac{1}{2}$. Therefore

$$\eta(\Gamma) = \begin{cases} e^{-i\pi/6} & JS_0^z < 0 \\ e^{i\pi/6} & JS_0^z > 0 \end{cases}. \tag{17}$$

K point.—We start by defining the states

$$|K, \nu, s\rangle = \frac{1}{\sqrt{6}} \sum_{j=0}^{5} e^{\frac{-i\pi}{6} j(2\nu+1)} \hat{C}_6^j \, |K, s\rangle \qquad \nu = 0, 1, \ldots, 5. \tag{18}$$

The six eigenspaces can be labelled by ν:

$$\hat{C}_6 \, |K, \nu, s\rangle = e^{\frac{i\pi}{6}(2\nu+1)} \, |K, \nu, s\rangle$$

The first-order effective 2×2 Hamiltonian in each eigenspace can be written $[H_\nu^{\text{eff.}}]_{ss'} = J \langle K, \nu, s | \vec{\sigma} \cdot \vec{S} | K, \nu, s' \rangle$, giving

$$H_0^{\text{eff.}} = H_3^{\text{eff.}} = J \begin{pmatrix} S_0^z + 2S_{\mathbf{G}}^z & 0 \\ 0 & -S_0^z + S_{\mathbf{G}}^z \end{pmatrix}, \tag{19a}$$

$$H_1^{\text{eff.}} = H_4^{\text{eff.}} = J \begin{pmatrix} S_0^z - S_{\mathbf{G}}^z & 0 \\ 0 & -S_0^z + S_{\mathbf{G}}^z \end{pmatrix}, \tag{19b}$$

$$H_2^{\text{eff.}} = H_5^{\text{eff.}} = J \begin{pmatrix} S_0^z - S_{\mathbf{G}}^z & 0 \\ 0 & -S_0^z - 2S_{\mathbf{G}}^z \end{pmatrix}. \tag{19c}$$

Let

$$\mu_K = \min[\pm J(S_0^z - S_{\mathbf{G}}^z), \pm J(S_0^z + 2S_{\mathbf{G}}^z)]. \tag{20}$$

It follows that

$$\theta(K) = \begin{cases} e^{i\pi/3} & \mu_K = J(S_0^z + 2S_{\mathbf{G}}^z) \\ e^{-i\pi/3} & \mu_K = -J(S_0^z + 2S_{\mathbf{G}}^z) \\ -1 \text{ or } e^{-i\pi/3} & \mu_K = J(S_0^z - S_{\mathbf{G}}^z) \\ e^{i\pi/3} \text{ or } -1 & \mu_K = -J(S_0^z - S_{\mathbf{G}}^z) \end{cases}. \tag{21}$$

The latter two cases require 2nd order perturbation theory, and thus higher Fourier harmonics, to resolve.

M point.—Define the states

$$|M, \nu, s\rangle = \frac{1}{\sqrt{6}} \sum_{j=0}^{5} e^{\frac{-i\pi}{6} j(2\nu+1)} \hat{C}_6^j \, |M, s\rangle \qquad \nu = 0, 1, \ldots, 5. \tag{22}$$

Once again the six eigenspaces can be labelled by ν:

$$\hat{C}_6 \left| M, \nu, s \right\rangle = e^{\frac{i\pi}{6}(2\nu+1)} \left| M, \nu, s \right\rangle . \tag{23}$$

The first-order effective Hamiltonian in each eigenspace is given by $[H_\nu^{\text{eff.}}]_{ss'} = \langle M, \nu, s | V | M, \nu, s' \rangle$, yielding

$$H_0^{\text{eff.}} = H_2^{\text{eff.}} = H_4^{\text{eff.}} = J \begin{pmatrix} S_0^z + S_{\mathbf{G}}^z & 0 \\ 0 & -S_0^z + S_{\mathbf{G}}^z \end{pmatrix}, \tag{24a}$$

$$H_1^{\text{eff.}} = H_3^{\text{eff.}} = H_5^{\text{eff.}} = J \begin{pmatrix} S_0^z - S_{\mathbf{G}}^z & 0 \\ 0 & -S_0^z - S_{\mathbf{G}}^z \end{pmatrix}. \tag{24b}$$

Let

$$\mu_M = \min[\pm J(S_0^z + S_{\mathbf{G}}^z), \pm J(S_0^z - S_{\mathbf{G}}^z)]. \tag{25}$$

It follows that

$$\zeta(M) = \begin{cases} i & \mu_M = J(\pm S_0^z + S_{\mathbf{G}}^z) \\ -i & \mu_M = J(\pm S_0^z - S_{\mathbf{G}}^z) \end{cases}. \tag{26}$$

Berry curvature

The profile of Berry curvature in k-space is essential for studies of strongly correlated physics in Chern bands. For example, a fractional Chern insulator is more strongly favored when the Berry curvature is flat (i.e. closer to the ideal case of a Landau level)[56]. Motivated by these concerns, we plot the Berry curvature for some textures explored in the main text in Fig. S4. We observe that at the "magic" magnetization $\bar{m} = 0.22$ where the lowest band becomes very flat, the Berry curvature becomes substantially more evenly distributed (than at $\bar{m} = 0.1$, for instance). The question of whether a fractional Chern insulator can be realized in this system is an interesting direction for future studies.

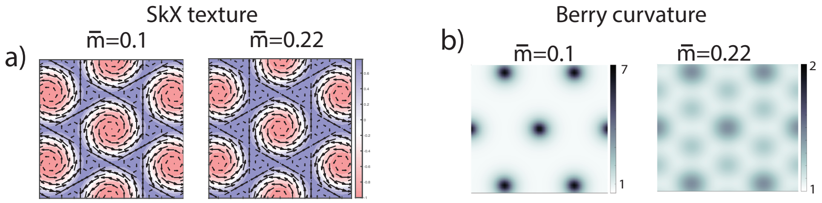

FIG. S4. **Berry curvatures for electrons coupled to skyrmion crystals.** (a) Skyrmion crystal textures reproduced from Fig. 2a. (b) Rescaled Berry curvature $|b_1|^2\Omega(\mathbf{k})/2\pi$ corresponding to the lowest bands in in Fig. 2b plotted in a square of size $2|b_1|$. Berry curvature becomes more uniform near the flat band at $\overline{m} = 0.22$.

REFERENCES AND NOTES

1. E. Y. Andrei, A. H. MacDonald, Graphene bilayers with a twist. *Nat. Mater.* **19**, 1265–1275 (2020).

2. T. T. Heikkilä, N. B. Kopnin, G. E. Volovik, Flat bands in topological media. *JETP Lett.* **94**, 233–239 (2011).

3. E. Tang and L. Fu, Strain-induced partially flat band, helical snake states and interface superconductivity in topological crystalline insulators. *Nat. Phys.* **10**, 964–969 (2014).

4. M. Serlin, C. L. Tschirhart, H. Polshyn, Y. Zhang, J. Zhu, K. Watanabe, T. Taniguchi, L. Balents, A. F. Young, Intrinsic quantized anomalous Hall effect in a moiré heterostructure, *Science* **367**, 900–903 (2020).

5. A. L. Sharpe, E. J. Fox, A. W. Barnard, J. Finney, K. Watanabe, T. Taniguchi, M. A. Kastner, D. Goldhaber-Gordon, Emergent ferromagnetism near three-quarters filling in twisted bilayer graphene. *Science* **365**, 605–608 (2019).

6. G. Chen, A. L. Sharpe, E. J. Fox, Y.-H. Zhang, S. Wang, L. Jiang, B. Lyu, H. Li, K. Watanabe, T. Taniguchi, Z. Shi, T. Senthil, D. Goldhaber-Gordon, Y. Zhang, F. Wang, Tunable correlated chern insulator and ferromagnetism in a moiré superlattice. *Nature* **579**, 56 (2020), 61.

7. T. Li, S. Jiang, B. Shen, Y. Zhang, L. Li, T. Devakul, K. Watanabe, T. Taniguchi, L. Fu, J. Shan, K. F. Mak, Quantum anomalous Hall effect from intertwined moiré bands. arXiv:2107.01796 (2021).

8. Y.-H. Zhang, D. Mao, T. Senthil, Twisted bilayer graphene aligned with hexagonal boron nitride: Anomalous Hall effect and a lattice mode. *Phys. Rev. Res.* **1**, 033126 (2019).

9. M. Xie, A. H. MacDonald, Nature of the correlated insulator states in twisted bilayer graphene. *Phys. Rev. Lett.* **124**, 097601 (2020).

10. F. Wu, T. Lovorn, E. Tutuc, I. Martin, A. H. MacDonald, Topological insulators in twisted transition metal dichalcogenide homobilayers. *Phys. Rev. Lett.* **122**, 086402 (2019).

11. T. Devakul, V. Crépel, Y. Zhang, L. Fu, Magic in twisted transition metal dichalcogenide bilayers. arXiv:2106.11954 (2021).

12. Y. Zhang, T. Devakul, L. Fu, Spin-textured Chern bands in AB-stacked transition metal dichalcogenide bilayers. *Proc. Natl. Acad. Sci. U.S.A.* **118**, e2112673118 (2021).

13. S. Mühlbauer, B. Binz, F. Jonietz, C. Pfleiderer, A. Rosch, A. Neubauer, R. Georgii, P. Böni, Skyrmion lattice in a chiral magnet. *Science* **323**, 915–919 (2009).

14. X. Z. Yu, N. Kanazawa, W. Z. Zhang, T. Nagai, T. Hara, K. Kimoto, Y. Matsui, Y. Onose, Y. Tokura, Skyrmion flow near room temperature in an ultralow current density. *Nat. Commun.* **3**, 988 (2012).

15. N. Nagaosa, Y. Tokura, Topological properties and dynamics of magnetic skyrmions. *Nat. Nanotechnol.* **8**, 899–911 (2013).

16. F. Büttner, I. Lemesh, G. S. D. Beach, Theory of isolated magnetic skyrmions: From fundamentals to room temperature applications. *Sci. Rep.* **8**, 4464 (2018).

17. Y. Machida, S. Nakatsuji, S. Onoda, T. Tayama, T. Sakakibara, Time-reversal symmetry breaking and spontaneous Hall effect without magnetic dipole order. *Nature* **463**, 210–213 (2010).

18. S. Nakatsuji, N. Kiyohara, T. Higo, Large anomalous Hall effect in a non-collinear antiferromagnet at room temperature. *Nature* **527**, 212–215 (2015).

19. A. K. Nayak, J. E. Fischer, Y. Sun, B. Yan, J. Karel, A. C. Komarek, C. Shekhar, N. Kumar, W. Schnelle, J. Kübler, C. Felser, S. S. P. Parkin, Large anomalous Hall effect driven by a nonvanishing berry curvature in the noncolinear antiferromagnet Mn_3Ge. *Sci. Adv.* **2**, e1501870 (2016).

20. Z. Liu, H. Wang, J. Wang, Magnetic moiré surface states and flat Chern bands in topological insulators. *Phys. Rev. B* **106**, 035114 (2022).

21. J. L. Lado, J. Fernández-Rossier, Quantum anomalous Hall effect in graphene coupled to skyrmions. *Phys. Rev. B* **92**, 115433 (2015).

22. P. Bruno, V. K. Dugaev, M. Taillefumier, Topological Hall effect and Berry phase in magnetic nanostructures. *Phys. Rev. Lett.* **93**, 096806 (2004).

23. A. Neubauer, C. Pfleiderer, B. Binz, A. Rosch, R. Ritz, P. G. Niklowitz, P. Boni, Topological Hall effect in the A phase of MnSi. *Phys. Rev. Lett.* **102**, 186602 (2009.

24. N. Paul, L. Fu, Topological magnetic textures in magnetic topological insulators. *Phys. Rev. Res.* **3**, 033173 (2021.

25. C.-K. Li, X.-P. Yao, G. Chen, Twisted magnetic topological insulators. *Phys. Rev. Res.* **3**, 033156 (2021).

26. S. Divic, H. Ling, T. Pereg-Barnea, A. Paramekanti, Magnetic skyrmion crystal at a topological insulator surface. arXiv:2103.15841 (2021).

27. G. Chen, M. Khosravian, J. L. Lado, A. Ramires, Designing spin-textured at bands in twisted graphene multilayers via helimagnet encapsulation. arXiv:2109.13130 (2021).

28. K. Hamamoto, M. Ezawa, N. Nagaosa, Quantized topological Hall effect in skyrmion crystal. *Phys. Rev. B* **92**, 115417 (2015).

29. Q. Tong, F. Liu, J. Xiao, W. Yao, Skyrmions in the moiré of van der Waals 2D magnets, *Nano Lett.* **18**, 7194–7199 (2018).

30. K. Hejazi, Z.-X. Luo, L. Balents, Noncollinear phases in moiré magnets. *Proc. Natl. Acad. Sci. U.S.A.* **117**, 10721–10726 (2020).

31. X. Lu, R. Fei, L. Zhu, L. Yang, Meron-like topological spin defects in monolayer $CrCl_3$. *Nat. Commun.* **11**, 4724 (2020).

32. M. Augustin, S. Jenkins, R. F. L. Evans, K. S. Novoselov, E. J. G. Santos, Properties and dynamics of meron topological spin textures in the two-dimensional magnet $CrCl_3$. *Nat. Commun.* **12**, 185 (2021).

33. Y. Xu, A. Ray, Y.-T. Shao, S. Jiang, D. Weber, J. E. Goldberger, K. Watanabe, T. Taniguchi, D. A. Muller, K. F. Mak, J. Shan, Emergence of a noncollinear magnetic state in twisted bilayer CrI3. arXiv:2103.09850 (2021).

34. D. Zhong, K. L. Seyler, X. Linpeng, R. Cheng, N. Sivadas, B. Huang, E. Schmidgall, T. Taniguchi, K. Watanabe, M. A. McGuire, W. Yao, D. Xiao, K. M. C. Fu, X. Xu, Van der Waals engineering of ferromagnetic semiconductor heterostructures for spin and valleytronics. *Sci. Adv.* **3**, e1603113 (2017).

35. T. P. Lyons, D. Gillard, A. Molina-Sánchez, A. Misra, F. Withers, P. S. Keatley, A. Kozikov, T. Taniguchi, K. Watanabe, K. S. Novoselov, J. Fernández-Rossier, A. I. Tartakovskii, Interplay between spin proximity effect and charge-dependent exciton dynamics in $MoSe_2/CrBr_3$ van der Waals heterostructures. *Nat. Commun.* **11**, 6021 (2020).

36. J. P. Perdew, K. Burke, M. Ernzerhof, Generalized gradient approximation made simple. *Phys. Rev. Lett.* **77**, 3865–3868 (1996).

37. H. Peng, Z.-H. Yang, J. P. Perdew, J. Sun, Versatile van der Waals density functional based on a metageneralized gradient approximation. *Phys. Rev. X* **6**, 041005 (2016).

38. G. Kresse and J. Furthmüller, Efficiency of ab-initio total energy calculations for metals and semiconductors using a plane-wave basis set. *Comput. Mater. Sci.* **6**, 15–50 (1996).

39. S. Haastrup, M. Strange, M. Pandey, T. Deilmann, P. S. Schmidt, N. F. Hinsche, M. N. Gjerding, D. Torelli, P. M. Larsen, A. C. Riis-Jensen, J. Gath, K. W. Jacobsen, J. J. Mortensen, T. Olsen, K. S. Thygesen, The computational 2D materials database: High-throughput modeling and discovery of atomically thin crystals. *2D Mater.* **5**, 042002 (2018).

40. M. Akram, H. LaBollita, D. Dey, J. Kapeghian, O. Erten, A. S. Botana, Moiré skyrmions and chiral magnetic phases in twisted CrX_3 (X = I, Br, and Cl) bilayers. *Nano Lett.* **21**, 6633–6639 (2021).

41. T. Song, Q.-C. Sun, E. Anderson, C. Wang, J. Qian, T. Taniguchi, K. Watanabe, M. A. McGuire, R. Stöhr, D. Xiao, T. Cao, J. Wrachtrup, X. Xu, Direct visualization of magnetic domains and moiré magnetism in twisted 2D magnets. *Science* **374**, 1140–1144 (2021).

42. K. S. Denisov, I. V. Rozhansky, N. S. Averkiev, E. Lähderanta, Electron scattering on a magnetic skyrmion in the nonadiabatic approximation. *Phys. Rev. Lett.* **117**, 027202 (2016).

43. K. Nakazawa, M. Bibes, H. Kohno, Topological Hall effect from strong to weak coupling. *J. Physical Soc. Japan* **87**, 033705 (2018).

44. K. S. Denisov, I. V. Rozhansky, N. S. Averkiev, E. Lähderanta, A nontrivial crossover in topological Hall effect regimes. *Sci. Rep.* **7**, 17204 (2017).

45. K. Karube, J. S. White, D. Morikawa, M. Bartkowiak, A. Kikkawa, Y. Tokunaga, T. Arima, H. M. Rønnow, Y. Tokura, Y. Taguchi, Skyrmion formation in a bulk chiral magnet at zero magnetic field and above room temperature. *Phys. Rev. Mater.* **1**, 074405 (2017).

46. J.-H. Park and J. H. Han, Zero-temperature phases for chiral magnets in three dimensions. *Phys. Rev. B* **83**, 184406 (2011).

47. Y. Tokura, N. Kanazawa, Magnetic skyrmion materials. *Chem. Rev.* **5**, 2857–2897 (2021).

48. S.-Z. Lin, S. Hayami, Ginzburg-landau theory for skyrmions in inversion-symmetric magnets with competing interactions. *Phys. Rev. B* **93**, 064430 (2016).

49. K. Shimizu, S. Okumura, Y. Kato, Y. Motome, Spin moiré engineering of topological magnetism and emergent electromagnetic fields. *Phys. Rev. B* **103**, 184421 (2021).

50. X. Z. Yu, Y. Onose, N. Kanazawa, J. H. Park, J. H. Han, Y. Matsui, N. Nagaosa, Y. Tokura, Real-space observation of a two-dimensional skyrmion crystal. *Nature* **465**, 901–904 (2010).

51. A. Tonomura, X. Yu, K. Yanagisawa, T. Matsuda, Y. Onose, N. Kanazawa, H. S. Park, Y. Tokura, Real-space observation of skyrmion lattice in helimagnet MnSi thin samples. *Nano Lett.* **12**, 1673–1677 (2012).

52. Y. Aharonov, A. Casher, Ground state of a spin-½ charged particle in a two-dimensional magnetic field. *Phys. Rev. A* **19**, 2461–2462 (1979).

53. S. L. Dudarev, G. A. Botton, S. Y. Savrasov, C. Humphreys, A. P. Sutton, Electron-energy-loss spectra and the structural stability of nickel oxide: An LSDA+ U study. *Phys. Rev. B* **57**, 1505–1509 (1998).

54. M. Wu, Z. Li, T. Cao, S. G. Louie, Physical origin of giant excitonic and magneto-optical responses in two-dimensional ferromagnetic insulators. *Nat. Commun.* **10**, 2371 (2019).

55. C. Fang, M. J. Gilbert, B. A. Bernevig, Bulk topological invariants in noninteracting point group symmetric insulators. *Phys. Rev. B* **86**, 115112 (2012).

56. S. A. Parameswaran, R. Roy, S. L. Sondhi, Fractional quantum Hall physics in topological flat bands. *C. R. Phys.* **14**, 816–839 (2013).
